# Biochemical Modification of Titanium Oral Implants: Evidence from In Vivo Studies

**DOI:** 10.3390/ma14112798

**Published:** 2021-05-24

**Authors:** Saturnino Marco Lupi, Mirko Torchia, Silvana Rizzo

**Affiliations:** Department of Clinical Surgical, Diagnostic and Pediatric Sciences, University of Pavia, 27100 Pavia, Italy; mirko.torchia01@universitadipavia.it (M.T.); srizzo@unipv.it (S.R.)

**Keywords:** surface properties, dental implant, coated materials, osseointegration

## Abstract

The discovery of osseointegration of titanium implants revolutionized the dental prosthesis field. Traditionally, implants have a surface that is processed by additive or subtractive techniques, which have positive effects on the osseointegration process by altering the topography. In the last decade, innovative implant surfaces have been developed, on which biologically active molecules have been immobilized with the aim of increasing stimulation at the implant–biological tissue interface, thus favoring the quality of osseointegration. Among these molecules, some are normally present in the human body, and the techniques for the immobilization of these molecules on the implant surface have been called Biochemical Modification of Titanium Surfaces (BMTiS). Different techniques have been described in order to immobilize those biomolecules on titanium implant surfaces. The aim of the present paper is to present evidence, available from in vivo studies, about the effects of biochemical modification of titanium oral implants on osseointegration.

## 1. Introduction

When a titanium implant is placed in bone tissue, its surface immediately reacts with several water molecules, creating a coat that surrounds the entire implant surface [1]. In a short time, it is covered by ions and biomolecules, followed by the non-specific adsorption of plasmatic proteins as a consequence of the so-called “Vroman effect”, which reaches an equilibrium between the phases of adsorption and desorption over time [2]. The entire process is influenced by implant surface properties, such as chemical composition, surface energy, and charge, that are determined prevalently by implant treatment processes during industrial production [3]. Protein layer stabilization on the implant surface enables interaction with the host tissue’s cells, which can recognize and bind proteins by cytoplasmic protrusions and membrane proteins. The specificity of cell–surface interaction is mainly due to the composition and organization of the protein layer, which, in turn, depends on the modalities with the implant surfaces earlier bonded water, ions, and biomolecules [1].

Based on the above, in the last decades increased research aimed at the functionalization of implant surfaces to obtain stimulatory effects on the biological tissues, to improve tissue response and, thus, increase the osseointegration process in addition to the long-term stability of the implant therapy. Initial attempts have been made mainly through the modification of surface topography, starting from the first machined titanium implants to get to the most recent micro- and nano-modified surfaces [4,5,6]. One of the most fascinating fields of research concerns the Biochemical Modification of Titanium Surfaces (BMTiS), defined by Puleo and Nanci in 1999 as a process that “utilize current understanding of the biology and biochemistry of cellular function and differentiation. (…) The goal of biochemical surface modification is to immobilize proteins, enzymes, or peptides on biomaterials for the purpose of inducing specific cell and tissue responses or, in other words, to control the tissue implant interface with molecules delivered directly to the interface. (…) Biochemical surface modification utilizes critical organic components of bone to affect tissue response” [7]. The purpose of implant surface functionalization by BMTiS derives from the supposition that the ability to imitate bone tissue characteristics may increment implant surface performances, thus, promoting the initial biological response [8]. Therefore, BMTiS, strictly speaking, refer only to the use of molecules normally present in the body; consequently, the authors of this review considered BMTiS in the strict sense and did not discuss all the techniques that involved the use of biochemical molecules not normally present in the body, such as, but not limited to, chitosan or pectin. In the implant dentistry field, studies concerning BMTiS have been carried out considering four main classes of biomolecules: (i) Peptides; (ii) Bone Morphogenetic Proteins (BMPs); (iii) non-BMPs growth factors; (iv) Extracellular matrix components (ECM). Their purpose is to promote specific adhesion and differentiation of osteogenic cells on the titanium implant surface, lending to improved osteogenic and osteoconductive properties [9].

The purpose of this review is to focus on the most recent advances obtained through the application of BMTiS in the field of dental implantology and to present the immobilization techniques of biomolecules and biomolecules on which research was carried out with particular attention to the results of in vivo studies.

## 2. Techniques That Can Be Used to Produce BMTiS

Different techniques to immobilize biomolecules on titanium implant surfaces have been described in the literature (Table 1).

### 2.1. Adsorption of Biomolecules by Immersion

Basically immersing the implant in a solution containing the selected biomolecule leads to physical adsorption and is one of the simplest techniques to immobilize biomolecules on implant surfaces. In this method interactions like Van der Waals or electrostatic forces ensure the bond between the two interfaces. Because of the weakness of those interactions, one of the limits of the technique is that it cannot handle the fixing and releasing processes of biomolecules on the implant surfaces [33]. As a consequence, the biomolecules that have been initially adsorbed can quickly detach from the surface. Liu et al., for example, show that simply adsorbed BMP-2 could be rapidly released by the implant surface [10]. The technique has also been exploited to immobilize other biomolecules such as non-BMPs growth factors [11,12,13,14,15,16,18,34], peptides [19,20,21], and ECM components [22,23,24,35,36].

### 2.2. Covalent Bonding

The covalent bonding technique determines a solid fixation of biomolecules on implant substrates. It is frequently employed to fix biomolecules such as ECM components on titanium implant surfaces [28]. The mechanism involves implant surface functionalization through amine, hydroxyl, and carboxyl groups that react with biomolecules and stabilize them to the surface.

The major advantage of the technique is the opportunity to fix a known amount of a determined biomolecule and to control its orientation on the substrate [37]. As reported by Xiao et al. [38], the main requirement is the preservation of the biological activity of bonded molecules, considering four crucial aspects: (A) Binding sites to substrate—that could be one or several depending on the reactive capabilities of the different molecules—must not interfere with the functional groups of the biomolecules; (B) To obtain the expected biological response, the distance between bioactive species and implant surface must be large enough to permit the interaction between the molecules and the surrounding environment; (C) Biomolecules fixed on the implant surface should not undergo denaturation or inactivation during the immobilization process, and should not be susceptible to variations when exposed to biological tissues; (D) The interaction between biomolecules and cellular receptors requires an adequate amount of reactive species on the surface. Bonded biomolecule density should not be excessive, to avoid molecular overcrowding reflecting a decrease in reactive abilities, but also should not be too low, indicating an inadequate cellular response stimulation.

To obtain covalent bonding, silanization represents the most common process to immobilize biomolecules on titanium implant surfaces; these are coated with silane or derivates molecules that work as a connection tool between the implant substrate and the biomolecules [39]. Concerning titanium oral implant surfaces, the present technique has been mainly employed to immobilize ECM components such as type I collagen [25,26,27] or hyaluronic acid, the latter both in animals [28] and humans [29].

### 2.3. Anodic Polarization

The high potential of anodic polarization can be exploited to augment the thickness of the oxide (TiO_2_) layer present on the implant surfaces, in increments up to 100 nm [3]. Anodic titanium-oxide ingrowth consists of a two-substrates system: the inner substrate corresponds to the metal/oxide interface, while the outermost corresponds to the oxide/electrolytes interface. The process occurs because of the reaction between metallic ions with water and electrolytes. Due to this interaction, ions, molecules, and nanoparticles previously adsorbed to the oxide/electrolytes interface may be incorporated into the growing anodic oxide layer [33]. As an example, Schliephake et al. exploited anodic polarization to immobilize collagen on titanium dental implant surfaces, obtaining positive effects on osseointegration [30].

### 2.4. Layer-by-Layer Technique (LBL)

One of the options to immobilize peptides or proteins exploits the deposition of molecular layers that bind electrostatically to the implant surface. The mechanism consists of the alternate adsorption of opposite-charged polyelectrolytes, which are stabilized because of attraction forces, creating a self-assembled polyelectrolytic multilayer (PEM) [40,41]. As the combination of PEMs and biomolecules can imitate native ECM and furnish adequate osteoconductive properties, LBL has been used to functionalize titanium surfaces by immobilizing organic bioactive molecules [42].

The immobilization on titanium dental implants of an adhesion peptide derived from laminin-5 using LBL, both in in vitro and in vivo studies, had the ability to improve soft tissue integration around the implant neck, which, according to the authors, may result in higher protection against peri-implant inflammation and better long-term stability [31].

Yang et al. employed the technique to immobilize peptides on titanium oral implant surfaces, finding positive effects on different osseointegration parameters in an in vivo animal model [32].

## 3. Biomolecules Used in In Vivo Studies

### 3.1. Peptides

The development of the tissues involves the adhesion of the cells to the extracellular matrix and the proliferation and organization of the matrix itself, giving rise to a functional evolution of the tissues [43,44].

In particular, a membrane receptor family known as integrins was identified for cellular adhesion to ECM [45]. Integrins react with short aminoacidic sequences present inside the matrix; among them, the Arg–Gly–Asp sequence (RGD sequence) plays a central role in mediating cellular adhesion to both plasmatic and ECM proteins, such as fibronectin, vitronectin, type I collagen, osteopontin, and bone sialoprotein [46]. Those simple aminoacidic sequences can be used to confer specific adhesion properties to the implant surfaces. As early as 1990, peptide bonding to biomaterials surface was studied to promote the interaction between surfaces and cells [47]. According to this approach, a single peptide with a weight of a few daltons (Da) can mediate cellular adhesion in a similar manner to that carried out by the same parental molecule, characterized by both higher dimensions and molecular weight. Since peptides can be synthetically produced, better control of their chemical composition is possible [9].

In the field of dental implantology, several studies have already experimented with the use of surface-bound peptides to modulate the characteristics of osseointegration. The results of the most significant studies are summarized in Table 2.

In a sample of miniature pig superior maxilla, the immobilization on Sandblasted Large grit Acid-etched (SLA) titanium implant surfaces of poly(L- lysine)-graft-poly(ethylene glycol) (PLL-g-PEG) bonded with RGD sequences (PLL-g-PEG/PEG-RGD) or Arg–Asp–Gly sequences (RDG sequences; PLL-g-PEG/PEG-RDG) resulted in significantly higher Bone–Implant Contact (BIC) values for PLL-g-PEG/PEG-RGD surfaces compared to non-coated SLA surfaces after 2 weeks of healing (61.68% against 43.62%; *p* < 0.001) [48]. Nevertheless, those differences were not significant after 4 weeks. Concerning PLL-g-PEG/PEG-RDG surfaces, significant differences with non-coated SLA surfaces have not been registered at either of the considered time intervals.

In a dog mandible model, the effects on osseointegration of a bioactive peptide containing aminoacidic sequences involved in bone development immobilized on titanium dental implant surfaces both at high and low concentrations were investigated [19]. In this study, the composition of the peptide used was not disclosed because it is protected as an industrial secret (Table 2).

In 2009, Yang et al. studied in a rabbit femur model of the effect on BIC values of RGD-sequences immobilized on titanium dental implant surfaces [32]. Histology was performed 4, 8, and 12 weeks after implant insertion, finding statistically significant differences between groups only at 4 weeks (23.25% against 16.75%; *p* < 0.05). In the same study, the authors also performed Removal Torque tests (RTQ) to evaluate the bone-implant contact strength. Results indicated after 8 and 12 weeks statistically significant differences between values registered in the two groups (*p* = 0.02 and *p* = 0.01 respectively) (Table 2). According to the authors, those results indicated a positive tendency of RGD-coated implants to bond bone tissue.

Furthermore, other peptides were investigated to study the effects on osseointegration of biochemical-modified titanium implant surfaces. In 2016, using a dog mandible model, Fiorellini et al. studied the effects on BIC of dental titanium implants coated by 200 μg/mL of recombinant human osteopontin (rhOPN) or by different concentrations (25, 50, 100, and 200 μg/mL respectively) of a synthetic, osteopontin-derived peptide (OC-1016), comparing the results with those obtained by control group samples (non-coated surfaces) [20]. A total of one hundred and forty-four implants were placed; data collection was performed after 4 and 12 weeks from surgery. At both time intervals, the authors registered significantly higher BIC values in all the coated groups than in the control group (*p* < 0.001 at 4 weeks and *p* < 0.05 at 12 weeks; Table 1). In the same study, the authors also evaluated newly formed bone density, finding at 4 weeks significantly higher values in coated groups than in the control group (*p* < 0.001; Table 1). On the contrary, at 12 weeks differences between groups were not significant.

VnP-16 is a vitronectin-derived peptide that has been demonstrated to promote bone apposition by stimulating osteoblastic activity and inhibiting osteoclasts [50].

In 2019, Cho et al., using a rabbit tibia model, evaluated the biofunctionalization of titanium dental implant surfaces with VnP-16 [21] (Table 2). The results, 14 days after surgery, indicated higher BIC values in VnP-16 coated implants, but not significantly. The newly formed bone area was also not significantly different between groups, although it was higher in VnP-16 coated implants than in the others.

More recently, Choi et al. studied the influence on osseointegration of an active, laminin-derived peptide (Ln2-P3) in a rabbit tibia model [49]. Laminin is an ECM protein mainly involved in the mechanism of adhesion and proliferation of basal lamina cells [51]. Authors equally divided 16 titanium dental implants characterized by SLA surface in two groups, according to the presence or absence of 1 mg/cm^2^ Ln2-P3 immobilized on the surface. After 9 healing days, histology revealed significantly higher BIC values for Ln2-P3-coated implants compared to the control group (56.2% against 23.4%; *p* = 0.01), although those differences were not significant after 11 days. Concerning newly formed bone area, the authors did not find significant differences between the values registered in the groups at either time interval (Table 2). The authors concluded that Ln2-P3 may play a role in osseointegration promotion mainly in the first healing stages.

### 3.2. Growth Factors

A growth factor (GF) is a protein that can promote mechanisms of replication, differentiation, and migration of certain cellular populations, with which they interact by specific membrane receptors. When a GF binds to a target cell’s receptor, it induces an intracellular signal transduction system that determines a precise biological response [52]. In the context of endosseous titanium implants, an increase of osteoblastic precursors and mesenchymal cell differentiation in active phenotype brings significant benefits to bone healing [53].

The major growth factors in bone tissue are as follows: Transforming Growth Factor β (TGF-β), Plasma-Rich Growth Factor (PRGF), acid and basic Fibroblast Growth Factor (aFGF, bFGF), and Insulin-like Growth Factor (IGF-I and IGF-II) [54,55]. The most important osteogenic GFs belong to the TGF-β superfamily, of which BMPs represent the most studied category, consisting of almost 18 different proteins [56]. Among them, BMP-2 possesses a high osteoconductive potential, which makes it widely used in the implant bio-functionalization field. Based on in vivo studies available in the literature, the two main forms of GF-BMP-2 and recombinant human BMP-2 (rhBMP-2) were studied in relation to osseointegration. The results of the most significant studies on BMTiS with GF are summarized in Table 2.

Concerning BMP-2, in a miniature pig superior maxilla model, both the adsorption or incorporation of BMP-2 on titanium, calcium phosphate implant surfaces (Ti + CaP + BMP-2) did not involve a significant increase in newly formed bone volume values after 3 weeks from surgery compared to Ti + CaP surfaces [10]. A similar study, in the same animal model, but considering newly formed bone volume values after 1, 2, and 3 weeks from surgery, confirmed non-significant differences between BMP-2 coated and non-coated Ti + CaP surfaces [12]. Authors stressed that the osteoconductive features of titanium implant surfaces may be negatively influenced by BMP-2 and in particular by the binding technique employed to immobilize it.

In a rabbit tibia and femur model, the immobilization on titanium implant surfaces of 800 mg of heparin combined with 50 ng/mL of BMP-2 or of the combination of hydroxyapatite, 800 mg of heparin, and 50 ng/mL of BMP-2, after 4 weeks of healing, did not reveal significant differences with hydroxyapatite-only coated and non-coated titanium surfaces in terms of BIC, RTQ, and newly formed bone volume (Table 3) [11].

Becker et al., in a dog mandible and tibia model, studied the effects on osseointegration of rhBMP-2 immobilized at different concentrations (596 and 819 ng/cm^2^ respectively) on titanium implant surfaces previously reinforced with Chromic-Sulfuric acid (CSA), and compared the obtained results with those collected by control group surfaces (Sand-blasted and Acid-etched) and CSA-reinforced surfaces [57]. BIC values registered both in tibia and mandible 4 weeks after surgery were found higher, but not significantly, in surfaces coated with the highest concentration of rhBMP-2, followed in decreasing order by those coated with 596 ng/cm^2^ of rhBMP-2, by CSA-reinforced surfaces, and by the control group ones. In the tibia model, the authors also performed bone density tests both at a distance inferior or superior to 1 mm from the surface, even in these cases without finding significant differences.

In a study by Lan et al., performed in a rabbit femur model, the immobilization of 1 mg of rhBMP-2 on titanium implant surfaces did not result in a bone formation increase after 4 and 8 weeks from implant placement if compared with control, non-coated surfaces (Table 3) [34]. On the contrary, pull-out tests performed at 12 healing weeks registered a significantly higher value for coated surfaces than for those of the control group (36.5 N against 27.6 N; *p* < 0.05).

Wikesjo et al., in a dog model, investigated the ability of rhBMP-2 to affect the osseointegration of titanium implants placed in the posterior mandibular region (type II bone) [15]. Titanium porous oxide (TPO) surfaces were coated with different rhBMP-2 concentrations (0.2 mg/mL and 4 mg/mL, respectively), and results, in terms of bone formation and BIC, were collected after 8 healing weeks and compared with those obtained by TPO non-coated surfaces. The results indicated higher bone formation, but not significantly, in coated implants. For BIC, non-coated surfaces have registered higher values compared to those obtained from coated surfaces (Table 3); however, in this case, the differences between groups were also not significant. A similar study, carried out in an adult monkey model, 16 weeks after implant placement in the molar region of the superior maxilla (type IV bone), evaluated the effects on bone formation and BIC of TPO surfaces coated with different concentrations of rhBMP-2, and compared the results with those obtained by non-coated TPO surfaces [16]. Even in that study, bone formation values were higher for coated implants than for non-coated ones, but not significantly. BIC values, on the contrary, were significantly higher for non-coated TPO surfaces (74.4%) than in TPO + 0.2 mg/mL rhBMP-2 (36.6%; *p* < 0.05) and in TPO + 2 mg/mL rhBMP-2 (43%; *p* < 0.01). Authors underlined that while for non-coated TPO surfaces peri-implant bone was primarily composed of residual native bone, in TPO + 2 mg/mL rhBMP-2 surfaces it was mainly represented by newly formed tissue.

Huh et al. used a form of rhBMP-2 derived from *E. Coli*—ErhBMP-2—to study the influence of that biomolecule on different parameters such as BIC, bone density between threads, and Implant Stability Quotient (ISQ), the latter obtained by Resonance Frequency Analysis (RFA) [13]. Anodized, non-coated surfaces and anodized, ErhBMP-2 coated surfaces were tested 8 weeks after surgery in a dog mandible. Concerning BIC, ErhBMP-2 immobilization did not result in significantly higher values compared to non-coated surfaces, as well as in the case of bone density between threads (Table 3). Otherwise, ISQ resulted in a significantly higher value in the ErhBMP-2 group compared to that obtained from the non-coated surface (79.21 against 74.27; *p* < 0.05).

In a rabbit tibia model, it was evaluated if non-coated, anodized titanium dental implant surfaces show significant differences in BIC values compared to the same surfaces coated by 80 μL poly(lactide-co-glycolic)acid (PLGA) + 50 μg/mL rhBMP-2 [58]. Bone-implant contact values have been registered after 3 and 7 healing weeks. BIC calculated over the entire length of the fixture had higher values, but not significantly, for coated implants both at 3 and 7 weeks. On the other hand, BIC values calculated after 3 weeks but confined to the first three threads of each implant indicate that statistically significant differences between coated and non-coated implants exist (67.59% against 46.08% at 3 weeks; *p* < 0.05).

Different studies available in the literature were carried out to evaluate the potential effects on osseointegration of the major bone tissue’s non-BMPs growth factors, evidencing different effects on peri-implant bone formation. Anitua studied, in a goat tibia and radius model, the effects on BIC values of PRGF immobilized on titanium dental implant surfaces, obtaining from the histomorphometric analysis at 8 weeks a statistically significant difference between the control group and the coated group (respectively, 21.89% against 51.28%; *p* < 0.01) [18].

In a rabbit femur model, the effect on the peri-implant bone formation of the immobilization of 1 mg of rhBMP-2, alone and in combination with 200 µg of recombinant human bFGF (rhbFGF) or 250 µg of recombinant human IGF-1 (rhIGF-1) was evaluated. A total of sixty-four implants were placed; to evaluate the bone formation and fluorescent bone markers were administered at 4 and 8 weeks [17]. Results obtained by the quantification of those markers at both time intervals revealed a higher, but not significantly, peri-implant bone formation in surfaces coated by the combination of rhBMP-2 with rhIGF-1 or rhbFGF than in the others (Table 4).

Concerning bFGF, Lee et al. showed, in a rabbit tibia model, that 100 ng of that biomolecule, combined with 0.02 mL of PLGA and immobilized on anodized titanium implant surfaces, involved significantly higher BIC values after 12 healing weeks compared to those obtained by anodized, non-coated and anodized, PLGA-only coated surfaces (44.7% against 31.4 and 33.6% respectively; *p* < 0.05) (Table 4) [59].

The effect of Fibroblast Growth Factor-Fibronectin (FGF-FN) on osseointegration was studied by Park et al. in a rabbit tibia model. All the tested implant surfaces were subjected to anodization, and those belonging to the coated group were also submerged for 24 h in a solution containing 65 µg/mL of FGF-FN [60]. Histology was conducted 12 weeks after surgery, finding BIC values significantly higher in FGF-FN surfaces (36.91%) than in the control group (29.47%; *p* < 0.05). The authors also performed RTQ tests, again finding significantly higher values in the coated group than in the control group (44.8 Ncm against 37.6 Ncm; *p* < 0.05).

The effects of TGF-β1 coating on osseointegration were investigated by Nikolidakis et al. in a goat femoral condyle model [14]. Authors divided the tested surfaces into 3 groups: group 1, composed of non-coated, sand-blasted and acid-etched surfaces; group 2, composed of surfaces identical to those of group 1, but coated with 0.5 µg of TGF-β1; group 3, composed of surfaces identical to those of group 1, but coated with 1 µg of TGF-β1. BIC values were collected after 6 healing weeks; surfaces belonging to group 3 registered significantly lower values if compared with those of group 1 (45% against 65%; *p* < 0.05). Even the surfaces of group 2 registered lower BIC values than those of group 1 (48% against 65%), although, in this case, the differences were not statistically significant. The authors concluded that low-dose TGF-β1 could have negative effects on the early stages of peri-implant bone healing.

Concerning recombinant human VEGF (rhVEGF), Schliephake et al., in a rat tibia model, evaluated if the combination of rhVEGF and anchor oligonucleotides immobilized on a sand-blasted and acid-etched surface (SA) (SA + ODN-AS + rhVEGF) was able to provide better results in terms of BIC values compared to non-coated SA surfaces and SA surfaces coated only with anchor oligonucleotides (SA + ODN-AS) [61]. Data collection was performed 1, 4, and 13 weeks after implant placement. At 4 weeks BIC values registered in SA + ODN-AS + rhVEGF group (60.1%) were significantly higher compared with the non-coated group and SA+ODN-As group (40.6 and 40.2% respectively; *p* = 0.008). On the contrary, at 1 and 13 weeks significant differences between groups were not registered (Table 4).

### 3.3. Extracellular Matrix Components

The extracellular matrix is a major component of the cellular environment, and it is mainly formed by collagen, glycoproteins, proteoglycans, and glycosaminoglycans, organized to create a highly site-specific system [35]. ECM is not only actively involved in osteoblast adhesion mediation, but also in mechanisms of migration, proliferation, and morphologic evolution and in the expression of specific genes. Therefore, the immobilization of the ECM components on the implant surface should mimic the native interface and influence the osteoblastic activity, increasing the biological response. [52]. Among the ECM components immobilized on titanium implant surfaces, type I collagen showed promising evidence both for its fundamental structural function in bone tissue and for the well-known role of osteoblast’s functions mediator in the cellular processes of adhesion, differentiation, and in ECM secretion [62].

Anodized, type I collagen-coated titanium surfaces registered in a rabbit tibia model higher BIC values compared to the same non-coated surfaces 4 weeks after implant placement (63.7% against 36.9%; *p* < 0.05) [20].

In a study carried out in a dog mandible model, the combination of type I collagen and calcium phosphate immobilized on machined titanium implant surfaces resulted in higher BIC values compared to the same, non-coated surfaces after 1 month of healing (62.6% against 31.5%; *p* < 0.05) [30]. Even the newly formed bone density test was performed at the same time interval, showing again significantly higher results in coated surfaces than in non-coated (40.9% against 16.1%; *p* < 0.05). In the same test, also collagen-only coated surfaces registered significantly higher results if compared to non-coated surfaces (33% against 16.1%; *p* < 0.05). BIC and density tests were repeated 3 months after implant placement, providing results in accordance with those registered at 1 month: both BIC and bone density values were significantly higher for coated implants compared with those collected in non-coated surfaces (Table 5).

In a rabbit tibia and femur model, 2 weeks after implant placement, acid-etched and type I collagen-coated titanium surfaces showed significantly better BIC values if compared to the non-coated surfaces (*p* = 0.028 for implants placed in tibia and *p* = 0.04 for those placed in the femur) [25]. On the contrary, 4 weeks after implant placement, significant differences were not registered. Acid-etched, type I collagen-coated surfaces were compared in terms of BIC values with non-coated surfaces also by Sverzut and Coll. in a dog mandible model [26]. BIC values after 3 and 8 healing weeks were not significantly different in the two groups. In that study were also evaluated newly formed bone area between threads (BABT) and within mirror area (BAMA), evidencing higher, but not significantly, results for coated implants (Table 5).

In a rabbit femur model, Scarano et al. studied the effects on osseointegration of type I collagen immobilized on sand-blasted and acid-etched titanium implant surfaces comparing the results with non-coated surfaces [27]. Data collection was performed 15, 30, and 60 days after surgery. At all the considered time intervals, BIC values were significantly higher for coated surfaces than for non-coated (Table 5). In that study, the authors also evaluated the newly formed bone area inside implant threads (BAIT) and outside them (BAOT), finding again significantly higher values in coated implants—within all considered time intervals—compared to non-coated surfaces (Table 5).

The effects of collagen on osseointegration parameters were also investigated by different authors in combination with glycosaminoglycans (GAGs) or GF. Given that the incorporation of chondroitin-sulfate (CS) in a collagen matrix can promote its interaction with GF and, therefore, simulate the ossification process, different studies evaluated the behavior of the collagen–CS matrix coated titanium implant surfaces [63]. In a study by Stadlinger et al., in a miniature pig mandible, collagen–CS coated surfaces registered slightly higher BIC values compared with those collected in the other tested groups (collagen-only coated surfaces and collagen-CS + rhBMP-4 coated surfaces) 22 weeks after implant placement [24]. Nevertheless, differences between groups were not significant. In the same study, the ISQ obtained from RFA tests in the two groups was not significantly different. A similar study, carried out in the same animal model but considering a healing period of 6 months, showed significantly higher BIC values for collagen-CS coated surfaces compared to those obtained by collagen-CS + rhBMP-4 (40% against 27%; *p* < 0.05), but not with those collected from collagen-only surfaces (40% against 30%) [35]. The authors indicated that the addiction of rhBMP-4 negatively affects the synergic activity of collagen and CS in promoting osseointegration. In a dog mandible model, 4 weeks after implant placement, the immobilization of collagen and of the combination of collagen with CS and rhBMP-2 (coll/CS/BMP-2) resulted in significantly higher BIC values compared to the control group (40.4 and 43.7% respectively against 25.4%; *p* < 0.05). On the contrary, at 12 weeks only the coll/CS/BMP-2 group and coll/CS group showed significantly higher BIC values compared to control samples (60.6 and 66.41% against 37.7%; *p* < 0.05).

In a miniature pig mandible model, 1 month after surgery, sand-blasted and acid-etched titanium implant surfaces coated with collagen and low and a high dose of CS showed significantly higher BIC values compared with the non-coated surfaces (68.4% for collagen + low dose CS against 51.6%; *p* < 0.001 and 63.1% for collagen + high dose CS against 51.6%, *p* = 0.0087) [22]. At two months, on the contrary, no significant differences were registered. Concerning RFA analysis, both at 1 and 2 months the ISQ values were not significantly different. In a systematic review and meta-analysis by Kellesarian et al., 72% of the 18 considered studies reported positive effects on bone formation, BIC values, and bone density, concluding that collagen-CS matrix immobilized on implant surfaces may improve the osseointegration process, thus supporting the prospective to perform phase I clinical trials in humans [64]. Nevertheless, as reported by the authors, further studies were required to define the precise dosage to obtain beneficial effects.

In a pig frontal bone model, the combination of 10 µg of type I collagen with 1 or 10 µg of BMP-2 or VEGF-165 or FGF-2, other than the combination of 1 or 10 µg of all those factors with 10 µg of type I collagen, did not reveal significant differences with non-coated surfaces 2 weeks after surgery [65]. On the contrary, at 4 weeks, type I collagen-only surfaces and type I collagen combined with 1 or 10 µg of VEGF-165 or 1 µg of FGF-2 surfaces registered significantly higher BIC values compared with non-coated groups (Table 6). At 8 weeks after implant placement, no significant differences were observed between groups.

Together with type I collagen and its combinations with GAGs or GF, in the BMTiS field, other ECM components or derivates were also investigated in terms of effects on osseointegration. Raphel et al. tested the efficacy of a coating composed of an elastin-like protein (ELP) and abundant in RGD sequences to promote cellular adhesion (RGD ELP) in rat tibia and femur, comparing it with the results obtained by scrambled-ELP coated surfaces and non-coated surfaces [66]. Histology and RTQ tests were performed respectively at 1, 4, and 8 weeks after implant placement. Results after 1 week indicated for RGD ELP-coated surfaces BIC values 70% larger than those obtained from the scrambled-ELP group, and 45% larger than those from the non-coated group, although in this latter case the differences were not significant because of the small sample size considered. Even at 4 and 8 weeks, significant differences between groups were not observed. Concerning the RTQ test, at 1 week, RGD ELP surfaces registered results 84% larger, but not significantly, than those obtained by non-coated surfaces. On the contrary, compared to the scrambled-ELP group, RTQ values registered in RGD ELP implants were significantly higher (*p* = 0.03); at 4 and 8 weeks, differences were not significant.

In a dog mandible model, Chang et al. studied the influence on implant osseointegration of a fibronectin coating, comparing the results with those obtained by the same, non-coated surfaces [67]. RFA tests performed at 2 and 4 weeks after surgery revealed significantly higher ISQ values for coated implants compared to non-coated (*p* < 0.01). On the contrary, at 8 weeks, differences between groups were not significant.

Due to its biological functions, hyaluronic acid has also been employed as a dental titanium implant coating. Morra et al. covalently linked 800-kDa hyaluronan on titanium implant surfaces (HATi), and tested its effects on different osseointegration parameters in a rabbit tibia model, comparing the results with those obtained from non-coated surfaces [28]. Significantly higher BIC values were registered in HATi surfaces compared to non-coated surfaces, both in cortical bone (69.7% against 55%; *p* < 0.05) and, particularly, in the trabecular one (69% against 22.5%; *p* < 0.01). Push-out test results for HATi surfaces were 25% higher than those collected in the control group (232.2 N against 185.3 N). In trabecular bone, the newly formed bone area was significantly higher for HATi surfaces than for non-coated ones (56.3% against 30.3%; *p* < 0.05). On the contrary, in cortical bone, statistically significant differences were not registered. These results could be due to the greater biological capacities of the trabecular bone compared to the cortical one. A newly formed bone hardness test was even performed, finding Bone Mineralization Index (BMI) values significantly higher for HATi surfaces than for the control ones (90.6% against 79.1%; *p* < 0.05) (Table 7).

In the literature, the only trial involving BMTiS conducted in humans was conducted by Lupi and Coll. proving the clinical non-inferiority of hyaluronan covalently coated titanium implant surfaces compared to the non-coated ones [29]. Marginal bone resorption values measured on intraoral radiographs both at mesial and distal implant sites did not reveal statistically significant differences between hyaluronan coated and non-coated surfaces until 36 months after surgery. The authors stressed the importance of conducting more detailed studies to evaluate hyaluronan benefits in more clinically complex cases, other than long-term follow up.

## 4. Conclusions

Biochemical modifications of titanium dental implants demonstrated benefits for the osseointegration process. Different techniques for the immobilization of biomolecules on titanium implant surfaces have been described, of which the most commonly used are physical adsorption, covalent bonding, anodic polarization, and LBL techniques. Different bioactive molecules were investigated for that purpose: concerning growth factors, the most frequently employed is rhBMP-2, but the effects of ErhBMP-2, PRGF, bFGF, IGF-1, FGF-FN, TGF-β1, and rhVEGF were also studied. Regarding peptides, the effects on osseointegration were considered for RGD sequences, rhOPN, OC-1016, VnP-16, and Ln2-P3. Among the ECM components, the most frequently used in the BMTiS field were type I collagen, alone or in combination with GF or chondroitin-sulfate, but the effects of fibronectin, an elastin-like protein (ELP) and hyaluronic acid, the latter both on animal and human models, were also studied.

On the basis of what has been discussed in the previous sections, the most significant applications of BMTiS involve the BIC parameter, which demonstrated the greatest benefits from the immobilization of biomolecules on implant surfaces. Moreover, when considered by the authors, even RTQ, ISQ, and newly formed bone density values provided positive evidence in favor of biochemically coated titanium implants [11,20,30,32,56,67].

Except for the mentioned randomized clinical trial about the usage of covalently-linked hyaluronan surfaces, work on the application of BMTiS in humans has not been found in the literature [29].

Based on the above, BMTiS represents a promising application for the characterization of titanium dental implant surfaces, although more studies are necessary, especially in humans, to confirm the evidence obtained in vivo on animal models.

## Figures and Tables

**Table 1:** Techniques to immobilize biomolecules on titanium implant surfaces.

Tecniques	Description	Biomolecules Reported from the Analyzed In Vivo Studies
Adsorption	Immersion or soaking of the implant in a solution containing the selected biomolecule	BMP-2 [10], non-BMPs growth factors [11,12,13,14,15,16,17,18], peptides [19,20,21] and ECM components [22,23,24]
Covalent bonding	Implant surface functionalization through amine, hydroxyl, and carboxyl groups that react with biomolecules and stabilize them to the surface	ECM components such as type I collagen [25,26,27] or hyaluronic acid [28,29]
Anodic Polarization	Molecules and nanoparticles previously adsorbed to the oxide/electrolytes interface may be incorporated in the growing anodic oxide layer	collagen [30]
Layer-by-layer technique	Alternate adsorption of opposite-charged polyelectrolytes, which are stabilized because of attraction forces, creating a self-assembled polyelectrolytic multilayer	laminin-5 [31], peptides [32]

**Table 2:** Peptides: results from the considered studies.

Author (Year)	Biomolecule of Interest	Tested Surfaces	Animal Model	Time from Surgery	Results
Germanier et al. (2006) [48]	RDG sequences, RGD sequences	1: SLA (control) 2: SLA + PLL-g-PEG 3: SLA + PLL-g-PEG/PEG-RDG 4: SLA + PLL-g-PEG/PEG-RGD	Miniature pig superior maxilla	2 and 4 weeks	BIC (2 w): 1: 43.62% ^4^; 2: 55.94%; 3: 48.54%; 4: 61.68% BIC (4 w): NSD
Barros et al. (2009) [19]	Bioactive peptide	1: SA 2: SA + HA 3: SA + HA + low concentration (20 µg/mL) bioactive peptide 4: SA + HA + high concentration (200 µg/mL) bioactive peptide	Dog mandible	8 weeks	BIC: NSD NFB: NSD
Yang et al. (2009) [32]	FGF-FN	1: ANO 2: ANO + 65 µg/mL FGF-FN	Rabbit femur	4, 8 and 12 weeks	BIC (4 w): 1: 16.75% *; 2: 23.25% RTQ (4 w): NSD BIC (8 w): NSD RTQ (8 w): 2 > 1 * BIC (12 w): NSD RTQ (12 w): 2 > 1 *
Fiorellini et al. (2016) [20]	rhOPN, OC-1016	1: TPS 2: TPS + 200 µg/mL rhOPN 3: TPS +25 µg/mL OC-1016 4: TPS +50 µg/mL OC-1016 5: TPS +100 µg/mL OC-1016 6: TPS + 200 µg/mL OC-1016	Dog mandible	4 and 12 weeks	BIC (4 w): 1: 45% ^2–6^; 2: 65.6%; 3: 61.3%; 4: 64.9%; 5: 73.1%; 6: 70.3% NFB (4 w): 1: 54.5% ^2–6^; 2: 70.5%; 3: 67.9%; 4: 69.3%; 5: 76.9%; 6: 72.6% BIC (12 w): 1: 46.5% ^2–6^*; 2: 62.5%; 3: 58.3%; 4: 59.5%; 5: 62.9%; 6: 63.5% NFB (12 w): NSD
Cho et al. (2019) [21]	VpN-16	1: Machined 2: SLA 3: SLA + scrambled VpN-16 4: SLA + 1 mg/cm^2^ VpN-16	Rabbit tibia	2 weeks	BIC: NSD NFB: NSD
Choi et al. (2020) [49]	Ln2-P3	1: SLA 2: SLA + 1 mg/cm^2^ Ln2-P3	Rabbit tibia	9 and 11 days	BIC (9 d): 1: 23.4% **; 2: 56.2% BIC (11 d): NSD NFB (9 and 11 d): NSD

**ANO:** anodized; **BIC:** Bone-Implant Contact; **d**: days; **HA:** Hydroxyapatite; **Ln2-P3:** Active peptide derived from laminin; **NFB**: Newly formed bone; **NSD:** Non-Significant Differences between groups; **OC-1016:** Active peptide derived from osteopontin; **PLL-g-PEG:** Poly(L-lysine)-graft-Polyethylene Glycol; **RDG:** Peptidic sequence Arg-Asp-Gly; **RGD:** Peptidic sequence Arg-Gly-Asp; **rhOPN:** Recombinant human osteopontin; **RTQ:** Removal Torque; **SA**: Sand-blasted and acid-etched; **SLA:** Sand-blasted Large-grit Acid-etched; **TPS:** Titanium plasma-sprayed; **VpN-16:** Functional peptide derived from vitronectin; **w**: weeks; *: *p* < 0.05; **: *p* < 0.01; ^4^ *p* < 0.001 relative to group 4; ^2–6^: *p* < 0.001 relative to all groups from 2 to 6; ^2–6^*: *p* < 0.05 relative to all groups from 2 to 6.

**Table 3:** BMPs: results from the considered studies.

Author (Year)	Biomolecule of Interest	Tested Surfaces	Animal Model	Time from Surgery	Results
Becker et al. (2006) [57]	rhBMP-2	1: SA 2: CSA 3: CSA + rhBMP-2: 596 ng/cm^2^ 4: CSA + rhBMP-2: 819 ng/cm^2^	Dog mandible and tibia	4 weeks	BIC (mandible and tibia): NSD Bone density (≤1 mm from surface, tibia): NSD Bone density (>1 mm from surface; tibia): NSD
Lan et al. (2007) [34]	rhBMP-2	1: 1 mg rhBMP-2 2: Non-coated	Rabbit femur	4, 8 and 12 weeks	NFB (4 and 8 w): NSD Pull-out test (12 w): 1: 36.5 N; 2:27.6 N *
Liu et al. (2007) [10]	BMP-2	1: Non-coated 2: Ti + CaP 3: Ti + CaP + BMP-2 adsorbed 4: Ti + CaP + BMP-2 incorporated 5: Ti + CaP + BMP-2 adsorbed + incorporated 6: Ti + BMP-2 adsorbed	Miniature pig superior maxilla	3 weeks	NFB: NSD
Wikesjo et al. (2008a) [15]	rhBMP-2	1: TPO 2: TPO + rhBMP-2 0.2 mg/mL 3: TPO + rhBMP-2 4 mg/mL	Dog mandible—molar region	8 weeks	NFB: NSD BIC: NSD
Wikesjo et al. (2008b) [16]	rhBMP-2	1: TPO 2: TPO + rhBMP-2 0.2 mg/mL 3: TPO + rhBMP-2 2 mg/mL	Monkey superior maxilla–molar region	16 weeks	NFB: NSD BIC: 1: 74.4%; 2: 36.6% *; 3: 43% **
Huh et al. (2012) [13]	ErhBMP-2	1: ANO 2: ANO + ErhBMP-2	Dog mandible	8 weeks	BIC: NSD Bone density between threads: NSD ISQ: 1:74.27; 2:79.21 *
Hunziker et al. (2012) [12]	BMP-2	1: Non-coated 2: Ti + CaP 3: Ti + CaP + 10 µg BMP-2 adsorbed 4: Ti + CaP + 12.95 µg BMP-2 incorporated 5: Ti + CaP + BMP-2 adsorbed + incorporated 6: Ti + BMP-2 adsorbed	Miniature pig superior maxilla	1, 2, and 3 weeks	NFB (1 w): NSD NFB (2 w): NSD NFB (3 w): NSD
Yang et al. (2014) [32]	BMP-2	1: Ti non-coated 2: Ti coated by Hap 3: Ti coated by 800 mg Hep and 50 ng/mL BMP-2 4: Ti coated by Hap, 800 mg Hep and 50 ng/mL BMP-2	Rabbit femur and tibia	4 weeks	BIC: NSD RTQ: NSD NFB: NSD
Yoo et al. (2015) [58]	rhBMP-2	1: ANO 2: ANO + 80 µL PLGA + 50 µg/mL rhBMP-2	Rabbit tibia	3 and 7 weeks	BIC (3 w): NSD BIC (7 w):1: NSD

**ANO:** anodized; **BIC:** Bone-Implant Contact; **CaP:** Calcium Phosphate; **CSA:** Chromic-Sulfuric acid; **ErhBMP-2:** E. Coli-derived recombinant human Bone Morphogenetic Protein-2; **Hap:** Hydroxyapatite; **Hep:** Heparin; **ISQ:** Implant Stability Quotient; **NFB**: Newly formed bone; **NSD:** Non-Significant Differences between groups; **PLGA:** Poly(lactide-co-glycolic) acid; **rhBMP-2:** Recombinant human Bone Morphogenetic Protein-2; **RTQ:** Removal torque; **SA**: Sand-blasted and acid-etched; **TPO:** Titanium Porous Oxide; **w**: weeks; *: *p* < 0.05; **: *p* < 0.01.

**Table 4:** Non-BMPs growth factors: results from the considered studies.

Author (Year)	Biomolecule of Interest	Tested Surfaces	Animal Model	Time from Surgery	Results
Anitua (2006) [18]	PRGF	1: Non-coated 2: Coated with PRGF	Goat tibia and radius	8 weeks	BIC: 1: 21.89% **; 2: 51.28%
Lan et al. (2006) [17]	rhBMP-2 rhbFGF rhIGF-1	1: PLA 2: PLA + 1 mg rhBMP-2 3: PLA + 1 mg rhBMP-2 + 200 µg rhbFGF 4: PLA + 1 mg rhBMP-2 + 250 µg rhIGF-1	Rabbit femur	4 and 8 weeks	NFB (4 w): NSD NFB (8 w): NSD
Park et al. (2006) [60]	FGF-FN	1: ANO 2: ANO + 65 µg/mL FGF-FN	Rabbit tibia	12 weeks	BIC: 1: 29.47% *; 2: 36.91% RTQ: 1: 37.6 Ncm *; 2: 44.8 Ncm
Nikolidakis et al. (2009) [14]	TGF- β1	1: SA 2: SA + 0.5 µg TGF-β1 3: SA + 1 µg TGF-β1	Goat femoral condyle	6 weeks	BIC: 1: 65%; 2: 48%; 3: 45% *
Lee et al. (2010) [59]	bFGF	1: ANO 2: ANO + 0.02 mL PLGA 3: ANO + 0.02 mL PLGA + 10 ng bFGF 4: ANO + 0.02 mL PLGA + 100 ng bFGF	Rabbit tibia	12 weeks	BIC: 1: 31.4% *; 2: 33.6% *; 3: 37%; 4: 44.7%
Schliephake et al. (2015) [61]	rhVEGF	1: SA 2: SA + ODN-AS 3: SA + (ODN-AS + rhVEGF)	Rat tibia	1, 4 and 13 weeks	BIC (1 w): NSD BIC (4 w): 1: 40.6% ^3^; 2: 40.2% ^3^; 3: 60.1% BIC: NSD

**ANO:** anodized; **BIC:** Bone-Implant Contact; **FGF-FN:** Fibroblast Growth Factor—Fibronectin; **NFB**: Newly formed bone; **NSD:** Non-Significant Differences between groups; **ODN-AS:** Anchor oligonucleotides; **PLA:** Poly-lactic acid; **PLGA:** Poly(lactide-co-glycolic) acid; **PRGF:** Platelet-Rich Growth factor; **rhBMP-2:** Recombinant human Bone Morphogenetic Protein-2; **rhbFGF:** Basic recombinant human Fibroblast Growth Factor; **rhIGF-1:** Recombinant human Insulin Growth Factor-1; **rhVEGF:** Recombinant human Vascular Endothelial Growth Factor; **RTQ:** Removal torque; **SA**: Sand-blasted and acid-etched; **TGF-β1:** Transforming Growth Factor-β1; *: *p* < 0.05; ** *p* < 0.01; ^3^ *p* = 0.08 relative to group 3.

**Table 5:** Collagen: results from the considered studies.

Author (Year)	Biomolecule of Interest	Tested Surfaces	Animal Model	Time from Surgery	Results
Morra et al. (2006) [36]	Collagen	1: ANO 2: ANO coated with collagen	Rabbit tibia	4 weeks	BIC: 1: 36.9% *; 2: 63.7% NFB: NSD
Schliephake et al. (2006) [30]	Collagen	1: MAC 2: MAC + type I collagen 3: MAC + calcium phosphate 4: MAC + calcium phosphate + type I collagen	Dog mandible	1 and 3 months	BIC (1 m): 1: 31.5% ^4^; 2: 41.9%; 3: 45.2%; 4: 62.6% NFB (1 m): 1: 16.1% ^2–4^; 2: 22.9%; 3: 33%; 4: 40.9% BIC (3 m): 1: 41.2% ^2–4^; 2: 60.2%; 3: 61.7%; 4: 59% NFB (3 m): 1: 40.6% ^2–4^; 2: 62.6%; 3: 58.5%; 4: 67.3%
Morra et al. (2010) [25]	Collagen	1: AE 2: AE coated withtype I collagen	Rabbit tibia and femur	2 and 4 weeks	BIC (2 w):2 > 1 * BIC (4 w): NSD
Sverzut et al. (2012) [26]	Collagen	1: AE 2: AE coated with type I collagen	Dog mandible	3 and 8 weeks	BIC (3 w): NSD BABT (3 w): NSD BAMA (3 w): NSD BIC (8 w): NSD BABT (8 w): NSD BAMA (8 w): NSD
Scarano et al. (2019) [27]	Collagen	1: SA 2: SA coated with type I collagen	Rabbit femur	15, 30 and 60 days	BIC (15 d): 1: 22.4% *; 2: 27.5% BAIT (15 d): NSD BAOT (15 d): 1: 19% *; 2: 21.8% BIC (30 d): 1: 51.2% *; 2: 55.3% BAIT (30 d): 1: 28% *; 2: 39% BAOT (30 d): 1: 36% *; 2: 38% BIC (60 d): 1: 56.3% *; 2: 63.6% BAIT (60 d): 1: 35% *; 2: 42% BAOT (60 d): 1: 36% *; 2: 44%

**AE:** acid-etched; **ANO:** anodized; **BABT:** Bone Area Between Threads; **BAIT:** Bone Area Inside Threads; **BAMA:** Bone Area Mirror Area; **BAOT:** Bone Area Outside Threads; **BIC:** Bone-Implant Contact; **d**: days; **MAC**: machined; **m**: months; **NFB**: Newly formed bone; **NSD:** Non-Significant Differences between groups; **SA**: Sand-blasted and acid-etched; **w**: weeks; *: *p* <0.05; ^4^: *p* < 0.05 relative to group 4; ^2–4^: *p* < 0.05 relative to groups 2, 3 and 4).

**Table 6:** Combinations of collagen and glycosaminoglycans or growth factors: results from the considered studies.

Author (Year)	Biomolecule of Interest	Tested Surfaces	Animal Model	Time from Surgery	Results
Stadlinger et al. (2007) [35]	Collagen CS rhBMP-4	1: Coated with COL 2: Coated with COL and CS 3: Coated with type COL and CS and rhBMP-4	Miniature pig mandible	22 weeks	BIC: NSD ISQ: NSD
Stadlinger et al. (2008) [24]	Collagen CS rhBMP-4	1: Coated with COL 2: Coated with COL and CS 3: Coated with COL and CS and rhBMP-4	Miniature pig mandible	6 months	BIC: 1: 30%; 2: 40%; 3: 27% ^2^
Schliephake et al. (2009) [23]	Collagen CS RGD sequence srhBMP-2	1: Machined 2: DAE 3: DAE coated with RGD sequences 4: DAE coated with COL 5: DAE coated with COL and CS 6: coll/CS + rhBMP-2	Dog mandible	4 and 12 weeks	BIC (4 w): 1: 25.4% ^2,3,4,6^; 2: 40.9%; 3: 41.4%; 4: 40.4%; 5: 31.1%; 6: 43.7% BIC (12 w): 1: 37.7% ^3,5,6^; 2: 57.6%; 3: 59.4%; 4: 56.3%; 5: 66.4%; 6: 60.6%
Stadlinger et al. (2009) [22]	Collagen CS	1: SA 2: SA coated by COL and low dose CS 3: SA coated by COL and high dose CS	Miniature pigmandible	1 weeks and 2 months	BIC (1 w): 1: 51.6% ^2,3^; 2: 68.4%; 3: 63.1% BIC (2 m): NSD ISQ (1 and 2 m): NSD
Mueller et al. (2011) [65]	Collagen BMP-2 VEGF-165 FGF-2	1: AE 2: coated with 10 µg COL 3: 2 + 1 µg BMP-2 4: 2 + 10 µg BMP-2 5: 2 + 1 µg VEGF-165 6: 2 + 10 µg VEGF-165 7: 2 + 1 µg FGF-2 8: 2 + 10 µg FGF-2 9: 2 + 1 µg of BMP-2, VEGF-165 and FGF-2 10: 2 + 10 µg of BMP-2, VEGF-165 and FGF-2	Pig frontal bone	2, 4 and 8 weeks	BIC (2 w): NSD BIC (4 w): 1: 39.4% ^2,5,6,7^; 2: 50.4%; 3: 46.8%; 4: 46.9%; 5: 64.9%; 6: 68.5%; 7: 54.8%; 8:74.1%; 9: 43.2%; 10: 44.2% BIC (8 w): NSD

**AE**: acid-etched; **BMP-2**: Bone Morphogenetic Protein-2; **BIC**: Bone-Implant Contact; **COL**: type I collagen; **CS**: chondroitin sulfate; **DAE**: Doubly acid-etched; **FGF-2**: Fibroblast Growth Factor-2; **m**: months; **NSD**: Non-Significant Differences between groups; **rhBMP-4**: Recombinant human Bone Morphogenetic Protein-4; **SA**: Sand-blasted and acid-etched; **VEGF-165**: Vascular Endothelial Growth Factor-165; **w**: weeks; ^2^ *p* < 0.05 relative to group 2; ^2,3,4,6^ *p* < 0.05 relative to groups 2, 3, 4 and 6; ^3,5,6^ *p* < 0.05 relative to groups 3, 5 and 6; ^2,3^ *p* < 0.0001 relative to group 2 and *p* = 0.0087 relative to group 3; ^2,5,6,7^ *p* < 0.05 relative to groups 2, 5, 6 and 7.

**Table 7:** Other ECM components investigated: results from the considered studies.

Author (Year)	Biomolecule of Interest	Tested Surfaces	Animal Model	Time from Surgery	Results
Morra et al. (2009) [28]	Hyaluronic acid	1: Non-coated 2: Coated with 800-kDa HY	Rabbit tibia	4 weeks	BIC (cortical): 1: 55% *; 2: 69.7% BIC (trabecular): 1: 22.5% **; 2: 69% Push-out: NSD NFB (cortical): NSD NFB (trabecular): 1:30.3% *; 2: 56.3% BMI: 1: 79.1% *; 2: 90.6%
Raphel et al. (2016) [66]	Scrambled ELP RGD ELP	1: Non-coated 2: Coated with scrambled ELP 3: Coated with RGD-ELP	Rat tibia and femur	1, 4, and 8 weeks	BIC (1 w): 3 > 2 * RTQ (1 w): 3 > 2 * BIC (4 w): NSD RTQ (4 w): NSD BIC (8 w): NSD RTQ (8 w): NSD
Chang et al. (2016) [67]	Fibronectin	1: Non-coated 2: Coated with fibronectin	Dog mandible	2, 4, and 8 weeks	ISQ (2 and 4 w): 2 > 1 ** ISQ (8 w): NSD NFB (2, 4 and 8 w): NSD
Lupi et al. (2019) [29]	Hyaluronic acid	1: Non-coated 2: Coated with HY	Humans	36 months	Clinical non-inferiority of HY coated surfaces compared to the non-coated

**BIC**: Bone-Implant Contact; **BMI**: Bone Mineralization Index; **ELP**: Elastin-like protein; **NFB**: Newly formed bone; **RTQ**: Removal torque; **NSD**: Non-Significant Differences between groups; **ISQ**: Implant Stability Quotient; **HY**: Hyaluronic acid; **w**: weeks; *: *p* < 0.05; **: *p* < 0.01.

## Data Availability

Data is contained within the article.
